# Physiology, phylogeny, and LUCA

**DOI:** 10.15698/mic2016.12.545

**Published:** 2016-11-25

**Authors:** William F. Martin, Madeline C. Weiss, Sinje Neukirchen, Shijulal Nelson-Sathi, Filipa L. Sousa

**Affiliations:** 1 Institute for Molecular Evolution, Heinrich-Heine Universität Düsseldorf, Universitätstrasse 1, 40225 Düsseldorf, Germany.; 2 Instituto de Tecnologia Química e Biológica, Universidade Nova de Lisboa, 2780-157 Oeiras, Portugal.; 3 Department of Ecogenomics and Systems Biology, University of Vienna, Althanstrasse 14, 1090 Vienna, Austria.; 4 Computational Biology & Bioinformatics Group, Rajiv Gandhi Centre for Biotechnology (RGCB), Trivandrum, Kerala 695014, India.

**Keywords:** early evolution, autotrophy, geochemistry, acetogens, methanogens

## Abstract

Genomes record their own history. But if we want to look all the way back to life's beginnings some 4 billion years ago, the record of microbial evolution that is preserved in prokaryotic genomes is not easy to read. Microbiology has a lot in common with geology in that regard. Geologists know that plate tectonics and erosion have erased much of the geological record, with ancient rocks being truly rare. The same is true of microbes. Lateral gene transfer (LGT) and sequence divergence have erased much of the evolutionary record that was once written in genomes, and it is not obvious which genes among sequenced genomes are genuinely ancient. Which genes trace to the last universal ancestor, LUCA? The classical approach has been to look for genes that are universally distributed. Another approach is to make all trees for all genes, and sift out the trees where signals have been overwritten by LGT. What is left ought to be ancient. If we do that, what do we find?

Early evolution and the nature of the very first kinds of life are interesting topics. They concern the phase of Earth history where our most distant ancestors emerged from the elements on an otherwise lifeless planet. The questions of how the initial evolutionary transition — from inanimate to animate matter — might have happened and what the first kinds of life were like in terms of habitat and lifestyle are just plain interesting. People generally want to know about how things were in the past, including the most distant past. It is apparently part of human nature to wonder where we came from.

An important concept in very early evolution is the last universal common ancestor, LUCA for short, because it represents the organism, cell, thing, or chemical reaction, depending on one's concept of LUCA, from which all life forms we know are descended. Thoughts about the nature of LUCA abound in the literature and are immensely diverse; the search term 'last universal common ancestor' alone returns 188 articles since 1997 in standard literature databases. Diversity of thoughts on LUCA is partly due to the circumstance that when we, as scientists, conceptually delve as deep as LUCA in evolutionary history, we are not far removed from the topic of life's origin. Thoughts on the origin of life are even more diverse than on LUCA, with over 2200 articles in literature databases appearing with 'origin of life' as the query. How can one learn more about the biology of LUCA, the starting point of early evolution?

If we look around, there are presently only two ways to empirically approach early evolution: geology and genomes. A prominent geologist, Andy Knoll, likes to say "Earth records its own history" 1, which is spot-on. Geology can indeed tell us when life arose. The oldest sedimentary rocks, which are ca. 3.8 billion years of age, harbour traces for life in the form of light carbon isotopes, evidence for biological CO_2_ fixation at that time 2,3. But the presence of CO_2_ fixation, possibly even as far back as 4.1 Ga 4 does not tell us everything that we might want to know about early life. Indeed, plate tectonics and erosion have erased much of the Earth's recorded history, with truly ancient rocks being rare and their evidence for early life often being difficult to interpret. Nonetheless, the geochemical record does harbor evidence for physiological processes.

A problem arises, though, in that physiological processes among prokaryotes are not generally restricted to any particular phylogenetic group. A glaring exception to that rule are the cyanobacteria, who also infringe upon the rule that Earth records its own history, because since cyanobacteria have been around, they have been editing a lot of Earth’s recorded text with their waste product, oxygen 5. Outside of the cyanobacteria, phylogeny and physiology are decoupled by the reality of lateral gene transfer (LGT) among prokaryotes: sulfate reduction 6, anoxygenic photosynthesis 5, fermentations 7, and respirations 8 are distributed among many different prokaryotic lineages, but because of LGT, not because of differential loss: LUCA could not do everything, it can hardly have possessed a genome of Eden. One might interject that methanogenesis *is* restricted to a particular phylogenetic group, the methanogens, but new phylogenetic depictions of the 'tree of life' have methanogens basal among the archaea, with loss of methanogenesis in many independent groups 9,10, those losses corresponding to gene acquisitions from bacteria in some cases 11, thereby decoupling phylogeny from physiology in the methanogens, too, which no longer appear as a monophyletic group.

Curiously, genomes also record their own history. But lateral gene transfer (much like plate tectonics) and sequence divergence (much like erosion) have erased much of the evolutionary signal that the very first genomes on our planet contained. Nonetheless we can be sure that there was a time and a place and an environment where those very first genomes did exist. How can one harness genomes to find out more about what the first life forms were like, and how to get a better picture of LUCA?

In the modern era (since the discovery of archaea), the ribosomal RNA tree of life, or the three domain tree 12, has been the main starting point for inferences about the nature of LUCA. But as progress has accrued with genomes, three issues have come to the fore that bear on inferences of LUCA's gene set: i) the effects of lateral gene transfer on our picture of LUCA, ii) the question of whether the three domain tree is correct, and iii) the issue of how universally distributed genes need to be in order to trace to LUCA.

The LGT issue is fairly straightforward. One avenue of investigation into LUCA has been to see which, what kind of and how many genes are common to archaea, bacteria and eukaryotes (all three domains). All things being equal, and barring LGT, such genes would trace to LUCA. So by simply looking for gene presence, Ouzounis *et al*. 13 could attribute about 1000 genes to LUCA, if LUCA was taken as the common ancestor of prokaryotes, or up to 1400 genes, if eukaryotes were included and if one allowed for widespread gene loss and excluded LGT. But like earlier investigations 14 and later investigations 15, Ouzounins *et al*. 13 attributed all absences of genes among lineages descended from LUCA to differential loss. If genes were distributed across domains by LGT, rather than differential loss, then presence of a gene in all three domains (or in both prokaryotic domains) would not reflect presence in LUCA, it would just reflect transdomain LGT. If not identified and removed, LGT generates overestimates of LUCA's gene content. Kannan *et al.* 16 very clearly spelled out the problem that transdomain LGT introduces into the study of LUCA's genes, and they also explained why it is not trivial to circumvent the LGT problem. The real problem with transdomain LGT is not that it has been known for many years to be an issue in early evolution 17, rather the real issue is its prevalence in nature today and in the past. Phylogenetic studies spanning all genes from many hundreds of genomes uncover thousands of cases of transdomain LGT, mainly from bacteria to archaea 11,18. If such LGT cases are identified and filtered out, maybe a picture of LUCA will come into focus.

The influence of the three domain tree on the issue of LUCA is somewhat more complicated. Many investigators on the issue of LUCA have adhered strictly to the three domain tree, meaning that if one wants to address LUCA, one must first place a root somewhere on the three domain tree. Investigations of anciently duplicated genes 19,20 led to placement of the root on the bacterial branch 12. But even among proponents of the three domain tree, the bacterial root was not universally accepted. For example, there have been strong proponents of the view that, the three domain tree is correct, but its root should be on the eukaryotic branch, coupled with the view that LUCA was more similar to eukaryotes than it was to prokaroytes 21,22,23,24 — a line of inference that has led its proponents to argue that the term 'prokaryote' be banned from the literature altogether. Di Guilio 25 also argues that we should ban the use of the term prokaryotes, albeit on grounds that do not hinge upon arguments that the first cells were eukaryote-like. Such discussions result in suggestions for terms like acaryotes, akaryotes, arkarya, and syncaryote 26 to replace the very useful concepts of prokaryotes and eukaryotes, terms which the more physiologically minded among us 27 are (wisely, we think) unwilling to surrender.

While debates about LUCA and higher order microbial nomenclature have been brewing, something else far more threatening for the three domain tree has been gnawing on its trunk: the three domain tree apparently has the domain relationships wrong. Recently, a small revolution in deep phylogenetic views has occurred, with newer methods of phylogenetic inference and investigations based on broader sampling of archaeal lineages having brought forth a new view of domain relationships, in which the archaeal component of eukaryotes branches *within* the archaea, not *as a sister* to them 9,28,29,30,31,32. Jim Lake will be quick to point out that some people had been saying that for 30 years 33. Defenders of the three domain tree counter that there is no need to worry, the three domain tree will persist 34. But people keep on finding the new tree of domain relationships, which is currently being called the two domain tree 29. Lake 33 (1988) called it the eocyte tree but the name did not stick well. In the two domain tree — which incidentally fits very well with what some of us have been saying about eukaryote origin for a long time 35 — genes that trace to LUCA need not be present in eukaryotes at all. That is because in the two domain tree, eukaryote genomes arose from a very small sample of prokaryotic gene diversity, in the simplest case from the symbiotic association of two prokaryotic genomes in the form of an archaeal host with a bacterial symbiont, the ancestor of mitochondria and hydrogenosomes 36,37.

Related to the issue of the three domain tree is the issue of how universal gene distributions need to be to trace a gene to LUCA. Regardless of where the root is, one can still look for genes that trace to LUCA by virtue of the density of their distribution. If one is strict, requiring that genes be universally distributed across genomes, about 30-36 genes trace to LUCA 38,39,40; if one allows for a bit of loss, about 100 genes trace to LUCA 41; if one allows for a bit more loss, then about 500-600 genes trace to LUCA 42; and if we allow for a lot of loss, then we are redirected to the issue above, namely that presence/absence patterns might be due to transdomain LGT rather than to differential loss, such that simple presence of a gene in bacteria and one archaeon or vice versa 15 is not solid ground for saying that said gene was present in LUCA.

In addition, if LUCA's gene set is defined in such a way that has to include genes that are present in eukaryotes (by the criterium of being present in *three* domains), then we quickly end up with an inference of LUCA that had a glycolytic pathway 42 and that used oxygen as a terminal acceptor 23, because that is how most eukaryotes obtain their energy 43. But we know from physiology that the first free-living cells cannot have been chemoorganotrophs (satisfying their energy needs by the oxidation or disproportionation of reduced carbon compounds) because organics from space are nonfermentable substrates 44. We also know from physiology that the producers of oxygen, cyanobacteria, represent a bioenergetically very advanced stage in physiological evolution 45,46, and thus cannot have preceded LUCA to generate oxygen for it to breathe. We also know from physiology that the mitochondria of many eukaryotes do not require oxygen for ATP synthesis 36.

Aware of the foregoing, we recently undertook a phylogenetic investigation based upon the two domain tree in search of insights into LUCA that might illuminate its microbial lifestyle 47. Rather than looking for genes that are universally distributed (or nearly universally distributed), we looked for genes that trace to LUCA by virtue of being ancient. As our criterion for ancient, we looked for genes that are present in bacteria and archaea, but not because of LGT. This approach embraces the two domain tree, in which eukaryotes have nothing to do with life's origin, thereby excluding eukaryotes from the analysis. But how to exclude LGT? We looked for genes that fulfill two very simple criteria: i) the gene is present in two members each of two major groups of archaea and bacteria and ii) the domains are monophyletic. Genes that fulfill those criteria are unlikely to have a distribution that results from LGT.

In order to identify such genes, there is presently no obvious alternative to making all trees for all genes in all sequenced genomes and separating the wheat (the trees that show domain monophyly in the two domain tree) from the chaff (the trees that show archaea and bacteria interleaving). We have been making trees for large numbers of genes for some time 11,18,48,49,50. Trees for all genes are important because it has become evident that in prokaryotes, each gene has its own independent evolutionary history and that "trees of life", whether based on rRNA or the currently popular collection of ribosomal proteins 29,30,38 are not good proxies for what genes will be present in the rest of the genome and how those genes will be related to homologues from other genomes, because LGT is very prevalent among prokaryotes.

When we were done sorting the trees, what we found in our analysis were 355 genes that depict LUCA as an anaerobic autotroph that lived in a hot, gas-rich, metal-rich environment 47. Its inferred energy metabolism was dependent upon H_2_ and CO_2_, it could fix N_2_, it had a heavy dependence upon transition metals, its metabolism revealed an extremely prominent role for methyl groups, one electron transfers, radical reactions, and redox chemistry. Its carbon metabolism was based on the acetyl-CoA pathway, the oldest of the six known CO_2_ fixation pathways. It was capable of substrate level phosphorylation using the acetyl-CoA pathway and it could harness chemiosmotic potential. It had modified bases, mostly involving methylations, suggesting that not only LUCA, but also the genetic code arose in an environment where reactive methyl groups were abundant. Previous studies had uncovered little information about LUCA's physiology and habitat. That is probably because earlier studies had focused on genes that are universally distributed (or nearly so). We also found that the trees of genes that trace to LUCA implicate clostridia (which harbour many acetogens) and methanogens as the earliest-branching forms of bacteria and archaea respectively. That fits with the functions of the genes we found, because acetogens and methanogens have carbon and energy metabolism that depends upon H_2_ and CO_2_, they can fix N_2_, they have a heavy dependence upon transition metals, and their core physiology reveals an extremely prominent role for methyl groups, one electron transfers, radical reactions, and redox chemistry.

The results that we obtained fit very well with the idea that life arose in submarine hydrothermal vents and that the first cells were autotrophs that satisfy both their carbon and their energy needs from the reduction of CO_2_ with electrons from H_2_ 51,52,53. Notably, H_2_ is still continuously generated in modern hydrothermal vents today by the process of serpentinization 54, a spontaneous and exergonic geochemical reaction in which Fe^2+^ in oceanic crust reduces H_2_O to generate H_2_, which can reach many concentrations in vent effluent of many millimols per liter 55. We found no evidence for a role of photosynthesis in LUCA's physiology, in particular there was no evidence for ZnS-based photosynthesis in LUCA (a physiology that is unknown among modern life forms anyway), in contrast to the predictions of some other recent theories 56. Rather we found evidence linking LUCA to known forms of microbial physiology — acetogenesis and methanogenesis without cytochromes 57 — that are manifest among the strictest anaerobes 58,59, with evidence for a role of sulfur metabolism 60, and with a very important role for Fe, Ni, Mo, and Co, transition metals that play a central role in the metabolism of anaerobic autotrophs today.

Our recent findings depart from phylogeny-based views of LUCA germane to the three domain tree and uncover connections between modern microbial physiology and geochemical environments on the early Earth. Some will surely complain that 355 genes is not enough and that essential functions like lipid synthesis, amino acid and nucleotide biosyntheses are very poorly represented in LUCA's gene set. How can anything live without that? As we wrote, lack of such essential functions among LUCA's gene set could indicate i) that the missing genes unspectacularly underwent transdomain lateral gene transfer (LGT) post-LUCA and hence were filtered out by our method, ii) that some missing chemical components were provided by spontaneous abiotic syntheses during early Earth history, or iii) a combination thereof. Transdomain LGT is both normal and natural, and all theories for the origin of cells, without exception, require abiotic syntheses, hence we do not see any fundamental problems in that regard. There was a time on the early Earth when there was no life and there was a time when there was life. If we filter out the effects of 4 billion years of LGT — which is, in essence, what we did — a picture of LUCA emerges that represents something that was half-alive, an intermediate in the transition from rocks and water on a young, barren planet to something that could scratch a living out of gasses and mineral salts. For some reason, that sounds quite reasonable to us, others will surely disagree.

It is very interesting that acetogens and methanogens inhabit the crust today 10,61. Geochemists say that the convective currents of water that permeate the Earth's crust to drive serpentinization have been going on since there was water on Earth 62. Let us presume, just for a moment, that the first bacteria and archaea were acetogens and methanogens respectively. On an uninhabited planet, they have no competitors, and life multiplies quickly given ample growth substrates. The founders of their respective domains would have bubbled off into the ocean bottom waters to be spread around by currents and eventually to be introduced back into hydrothermal systems in the crust, where they would have found the diet that they were raised on. It is possible that some anaerobic autotrophs that live from the reduction of CO_2_ with H_2_ still inhabit the same *niche* in which life arose, albeit not the same *rocks* because during Earth history oceanic crust is constantly recycled into the mantle via subduction. In that sense, acetogens and methanogens really might provide a glimpse into the biology of the very first microbes on Earth, as some microbiologists familiar with the physiology of these organisms have been saying for some time 45,60,63.

Over four decades ago, biochemists thought that FeS clusters are ancient 64 and that acetogens and methanogens are ancient 45, based on good intuition, common sense, and some straightforward principles of physiology. With the discovery of archaea, the three domain tree led to avenues of thought about early evolution that were guided by phylogeny rather than physiology. LGT conflates phylogeny. But LGT does not conflate physiology, it just decouples it from phylogeny. When we filter out the LGT from all of the gene trees that we can make from genomes, we end up with a picture of LUCA that looks very much like what experts familiar with the physiology of anaerobes had in mind in the late 1960's 45, and still have in mind today 65,66. If we return to the geochemical record, the first evidence for life we see is evidence for autotrophs 3,4, which is also what genomes recently uncovered about LUCA 47. Thus, on the issue of autotrophs being ancient, geology and physiology converge. The version of LUCA that is obtained by taking all the data and simply removing the obvious LGT interfaces well with Earth history, with microbial physiology, and even with the new two domain tree. It also bears out the predictions of some specific formulations the theory that life arose at submarine hydrothermal vents.
